# Immune microenvironment in ductal carcinoma in situ: a comparison with invasive carcinoma of the breast

**DOI:** 10.1186/s13058-020-01267-w

**Published:** 2020-03-26

**Authors:** Milim Kim, Yul Ri Chung, Hyun Jeong Kim, Ji Won Woo, Soomin Ahn, So Yeon Park

**Affiliations:** 1 Department of Pathology, Seoul National University Bundang Hospital, 82, Gumi-ro 173 Beon-gil, Bundang-gu, Seongnam, Gyeonggi 13620 Republic of Korea; 2 Department of Pathology, Seoul National University College of Medicine, Seoul, Republic of Korea

**Keywords:** Ductal carcinoma in situ, Immune microenvironment, CD4, CD8, FOXP3, PD-L1

## Abstract

**Background:**

The immune microenvironment in ductal carcinoma in situ (DCIS) and its significance are not well established. This study was conducted to evaluate the immune microenvironment of DCIS including the composition of tumor-infiltrating lymphocyte (TIL) subsets and PD-L1+ immune cells and to compare it with that of invasive breast cancer.

**Materials and methods:**

A total of 671 cases including three different disease groups of pure DCIS, DCIS with microinvasion (DCIS-M), and invasive carcinoma were included in this study. CD4+, CD8+, and FOXP3+ TIL subsets and PD-L1+ immune cells were detected with immunohistochemistry using tissue microarrays and were analyzed in relation to clinicopathologic characteristics and different disease groups.

**Results:**

In pure DCIS, high infiltrations of CD4+, CD8+, and FOXP3+ T cells and the presence of PD-L1+ immune cells were associated with high nuclear grade, comedo-type necrosis, hormone receptor (HR) negativity, and high Ki-67 proliferation index. All immune cell infiltrations were higher in invasive carcinoma than in pure DCIS regardless of the HR status. While CD4+ T cells were more abundant than CD8+ T cells in pure DCIS, CD8+ T cells were dominant in invasive carcinoma, especially in HR-negative tumors. Within individual cases of invasive carcinoma with DCIS component, all immune cell subset infiltration was higher in the invasive component than in the DCIS component; however, CD4+ TIL infiltration did not differ between the two components in HR-negative tumors. Comparing pure DCIS, DCIS-M, and DCIS associated with invasive carcinoma (DCIS-INV), CD4+ TIL infiltration revealed a gradual increase from pure DCIS to DCIS-M and DCIS-INV in the HR-negative group, whereas FOXP3+ TIL infiltration was significantly increased in DCIS-INV than in pure DCIS in the HR-positive group. The high infiltration of FOXP3+ TIL and the presence of PD-L1+ immune cells were associated with tumor recurrence in patients with pure DCIS.

**Conclusions:**

Our study showed that the immune microenvironment differs significantly not only between DCIS and invasive carcinoma but also between pure DCIS, DCIS-M, and DCIS-INV depending on the HR status.

## Background

Ductal carcinoma in situ (DCIS) is an early pathologic stage of breast cancer characterized by proliferation of tumor cells within the ductal-lobular unit. Introduction of screening mammography has resulted in increased detection of DCIS which currently accounts for 20~25% of newly diagnosed breast cancers in the USA [1]. DCIS is a non-obligatory precursor of invasive breast cancer that progresses to invasive cancer over 10–15 years in 14–53% [2]. The mechanism by which DCIS progresses to invasive carcinoma is not well understood, but it is thought to be a complex process driven by tumor cells (through genetic aberrations or altered expression of genes critical for invasion) and tumor microenvironment including myoepithelial cells, stromal fibroblasts, and immune infiltrates [3].

The immune system can eliminate tumor cells or control tumor growth by immune surveillance, but interaction between tumor cells and the immune system is known to play a crucial role in tumor progression [4]. Key players of the immune system include myeloid cells, lymphocytes, cytokines, and chemokines, of which tumor-infiltrating lymphocytes (TILs) are thought to represent tumor immunogenicity, and their composition is associated with the direction of an immune response [5, 6]. Studies to date generally agree that CD8+ cytotoxic T lymphocytes (CTLs) and CD4+ Th1 cells are involved in effective anti-tumor immunity while FOXP3+ regulatory T cells are associated with suppression of anti-tumor immunity [7]. Besides TILs, immune checkpoint molecules are also involved in the regulation of anti-tumor responses. Especially, programmed death-ligand 1 (PD-L1), also known as B7-H1 or CD274, is expressed on tumor cells and immune cells; it suppresses T cell migration, proliferation, and secretion of cytotoxic mediators, and it also restricts tumor cell killing through binding to programmed death-1 (PD-1) and B7.1 (CD80) [8].

Most of the previous studies on TILs and their composition in breast cancer have focused on their predictive and prognostic significance in invasive breast cancer [9–14]; their presence and significance in DCIS remain elusive. A few studies have reported that dense TILs in DCIS were associated with more aggressive clinical features and an increased risk to progression [15, 16], and specific TIL subsets in DCIS have been linked to tumor recurrence [17–19]. However, there is a lack of studies on TIL subset infiltration in DCIS and its comparison with that of invasive breast cancer. Moreover, only a few studies have evaluated PD-L1+ immune cells in DCIS with a limited number of cases [16, 20–22]. Since interaction between tumor cells and its immune microenvironment play an essential role in tumor progression, evaluation of the differences in the infiltration of immune cell subsets between DCIS and invasive carcinoma may help us understand the mechanism for progression of DCIS.

Thus, in this study, we examined infiltrations of CD4+, CD8+, and FOXP3+ TILs and PD-L1+ immune cells in DCIS in relation to clinicopathologic features and compared them with those of invasive breast cancer to evaluate their changes during progression of DCIS.

## Methods

### Tissue samples

This retrospective study included a total of 671 cases comprising 231 cases of pure DCIS, 81 cases of DCIS with microinvasion (DCIS-M), and 359 cases of invasive breast carcinoma that had been diagnosed between 2003 and 2011 at Seoul National University Bundang Hospital. In a previous study, 377 cases of invasive breast carcinoma had been used for the evaluation of TIL subset infiltrations except for PD-L1 [14]. After excluding 18 cases of invasive lobular carcinoma, the data of the remaining 359 invasive carcinomas were used for this study. Of the 359 invasive carcinoma cases, ninety cases which had a sufficient amount of DCIS component associated with invasive carcinoma were selected for comparative analysis of the invasive and DCIS components within the same tumor. Clinicopathologic information was obtained by reviewing medical records and hematoxylin and eosin (H&E)-stained sections. For pure DCIS and DCIS-M, clinicopathologic data including the patient age, tumor extent, nuclear grade, presence of comedo-type necrosis, architectural pattern, presence of microinvasive foci, estrogen receptor (ER), progesterone receptor (PR), human epidermal growth factor receptor 2 (HER2) status, Ki-67 proliferation index, and p53 overexpression were recorded. For invasive carcinomas, clinicopathologic variables had been summarized in a previous study [14]. Baseline characteristics of pure DCIS and DCIS-M are summarized in Table 1. These two disease groups were significantly different in various clinicopathologic characteristics including DCIS extent, nuclear grade, ER, PR, and HER2 status.

**Table 1:** Clinicopathologic characteristics of pure ductal carcinoma in situ (DCIS) and DCIS with microinvasion

Clinicopathologic characteristics		Pure DCIS (*n =* 231)	DCIS with microinvasion (*n =* 81)	*p* value
Age (years)	Median (range)	47 (25–88)	48 (26–76)	0.556
Extent of DCIS (cm)	Median (range)	2.5 (0.4–12.2)	4.2 (0.9–14.5)	< 0.001
Nuclear grade	Low	16 (6.9)	2 (2.5)	< 0.001
	Intermediate	129 (55.8)	20 (24.7)	
	High	86 (37.2)	59 (72.8)	
Comedo-type necrosis	Absent	181 (78.4)	30 (37.0)	< 0.001
	Present	50 (21.6)	51 (63.0)	
ER	Negative	30 (13.0)	42 (51.9)	< 0.001
	Positive	201 (87.0)	39 (48.1)	
PR	Negative	44 (19.0)	47 (58.0)	< 0.001
	Positive	187 (81.0)	34 (42.0)	
HER2	Negative	196 (84.8)	43 (53.1)	< 0.001
	Positive	35 (15.2)	38 (46.9)	
Ki-67 index	< 10%	171 (74.0)	30 (37.0)	< 0.001
	≥ 10%	60 (26.0)	51 (63.0)	
P53	Negative	201 (87.0)	51 (63.0)	< 0.001
	Positive	30 (13.0)	30 (37.0)	

*p* values were calculated by the Mann-Whitney *U* test and chi-square or Fisher’s exact test. Numbers in parentheses indicate column percentage

*ER* estrogen receptor, *PR* progesterone receptor, *HER2* human epidermal growth factor receptor 2

### Tissue microarrays

H&E-stained slides from formalin-fixed, paraffin-embedded tissue blocks were reviewed, and representative sections were selected in each case for construction of tissue microarrays (TMAs). For pure DCIS and DCIS-M, one to three tissue columns of 4-mm-diameter circles (depending on the extent of the tumor) were arranged in TMA using a trephine apparatus (Superbiochips Laboratories, Seoul, Korea). For invasive carcinoma, three sets of TMAs (2 mm in diameter) that had been constructed for the previous study [14] were used. For comparative analysis between invasive and DCIS components of the same tumor, one tissue column of DCIS associated with invasive carcinoma (DCIS-INV) (4 mm in diameter) was selected and was made into TMAs.

### Evaluation of basic biomarkers

The expression of the basic biomarkers including ER, PR, HER2, p53, and Ki-67 was evaluated from the surgical specimens at the time of diagnosis. As for those with missing data, immunohistochemical staining on representative tissue sections was carried out using the following antibodies: ER (clone SP1; 1:100 dilution; LabVision, Fremont, CA), PR (clone PgR 636; 1:70 dilution; Dako, Carpinteria, CA), HER2 (clone 4B5; ready to use; Ventana Medical Systems, Tuscon, AZ), p53 (clone D07; 1:600 dilution; Dako), and Ki-67 (clone MIB-1; 1:250 dilution; Dako).

ER and PR were regarded as positive if at least 1% of the tumor cells were stained. HER2 positivity was defined as an immunohistochemical score of 3+ or the presence of gene amplification on fluorescence/silver in situ hybridization. For p53, staining in 10% or more of the tumor cells was considered positive. High Ki-67 proliferation index was defined as staining in 10% or more of the tumor cells.

### Immunohistochemistry for immune cells and counting

Immunohistochemical staining was performed with a BenchMark XT autostainer (Ventana Medical Systems) using an UltraView detection kit (Ventana Medical Systems) for CD4, CD8, FOXP3, and PD-L1. The following antibodies were used: CD4 (Clone SP35; ready to use; Dako), CD8 (Clone C8/144B; ready to use; Dako), FOXP3 (Clone 236A/E7; 1:100 dilution; Abcam, Cambridge, UK), and PD-L1 (clone E1L3N; 1:100 dilution; Cell Signaling, Danvers, MA).

For the evaluation of CD4+, CD8+, and FOXP3+ T cell infiltration, the number of each TIL subset was counted by two pathologists (MK and YRC) who were blinded to the clinicopathologic features of the tumors. From the TMA cores, three areas with the highest infiltration were chosen under the high-power field (400X), and the number of TIL subset was counted in both intra-tumoral and stromal compartments either manually or using a digital image analyzer, ScanScope CS system (Aperio, Vista, CA). Then, the average numbers of CD4+, CD8+, and FOXP3+ T cells from the selected areas were calculated. In cases of DCIS, the stromal compartment was defined as the area of the specialized stroma surrounding the involved ducts, or when it is not clear, as an area surrounding ducts within 2 high-power fields according to a proposal from the International Immuno-Oncology Biomarker Working Group [23, 24]. As for PD-L1, PD-L1+ immune cells were considered to be present when at least 1% of the tumor stromal area was occupied by PD-L1+ immune cells, as previously described [16].

### Statistical analysis

Statistical analysis was performed using Statistical package, SPSS version 25.0 for Windows (IBM Corp., ARMONK, NY). CD4+, CD8+, and FOXP3+ TIL counts did not show normal distributions, and thus, non-parametric tests were used for statistical analyses. Spearman’s rank correlation tests were used to assess the associations among infiltrations of CD4+, CD8+, and FOXP3+ TILs and PD-L1+ immune cells. The difference in the infiltration of CD4+, CD8+, and FOXP3+ TILs was analyzed by the Mann-Whitney *U* test between two groups and by the Kruskal-Wallis test among three groups. For the detection of the predominant TIL subset in the same disease group or for comparison of TIL subset infiltration between invasive and in situ components in the same tumor, the Wilcoxon signed rank test was used. The presence or absence of PD-L1+ immune cells was analyzed by chi-square test between groups, and its comparison between invasive and in situ components in the same tumor was performed using the McNemar test. In pure DCIS, a receiver operating characteristic (ROC) curve analysis was performed to identify the cut-off values for CD4+, CD8+, and FOXP3+ TILs and ratios of TIL subsets (FOXP3+/CD8+ TIL, FOXP3+/CD4+ TIL, and CD4+/CD8+ TIL) that maximized the sum of sensitivity and specificity in predicting ipsilateral breast cancer recurrence using recurrence as a dichotomous outcome. Recurrence-free survivals were analyzed by drawing Kaplan-Meier curves, and differences were determined with the log-rank test. When comparing immune cell subset infiltration among pure DCIS, DCIS-M, and DCIS-INV, corrections for multiple testing were made by the Bonferroni method, and adjusted (adj.) *p* values were calculated. *p* values less than 0.05 were considered significant with all reported *p* values being two-sided.

## Results

### Infiltration of immune cell subsets and their relationship with clinicopathologic features of pure DCIS

**Fig. 1:**
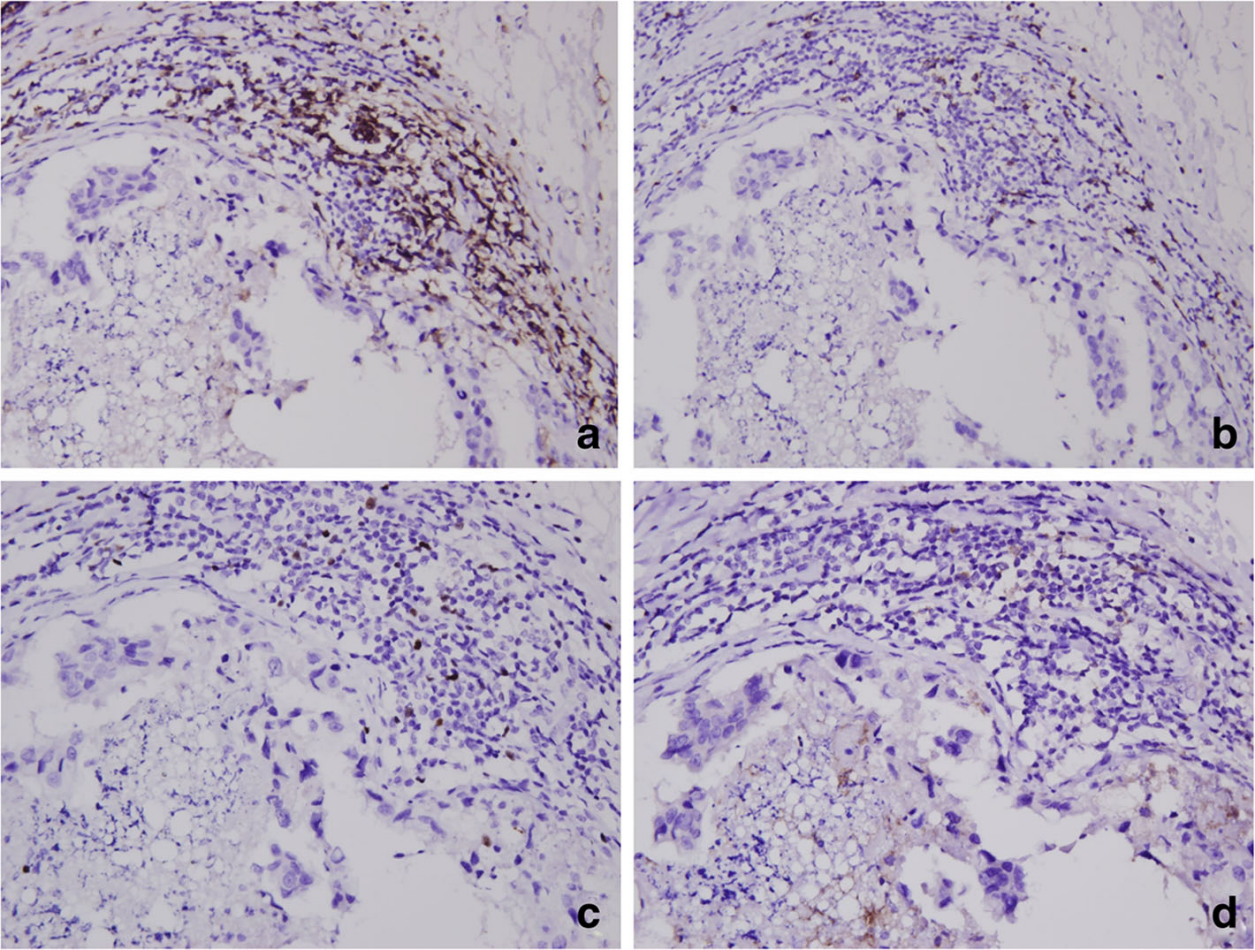
Representative example of CD4+, CD8+, and FOXP3+ tumor-infiltrating lymphocyte and PD-L1+ immune cell infiltration in pure ductal carcinoma in situ. CD4+ (**a**), CD8+ (**b**), and FOXP3+ tumor-infiltrating lymphocytes (**c**) and PD-L1+ immune cells (**d**) are predominantly found in the peri-tumoral specialized stroma around the involved ducts and are detected in rare numbers within tumor cell nests

In pure DCIS, immune cell infiltration was usually observed in the peri-tumoral specialized stroma surrounding the involved ducts (Fig. 1). CD4+, CD8+, FOXP3+ TILs and PD-L1+ immune cells were found within tumors in rare numbers. The infiltration of CD4+, CD8+, and FOXP3+ TILs was quite variable, and they were present in 90.2%, 99.1%, and 30.9% of total cases, respectively. The median numbers of CD4+ and CD8+ TILs were 17.8 (range, 0–279) and 12.5 (range, 0–171), respectively, per high-power field. The number of tumor-infiltrating FOXP3+ T cells ranged from 0 to 30 under the high-power field. PD-L1 expression in tumor cells was rarely observed with focal positivity in three cases of high-grade DCIS. PD-L1+ immune cells were observed in 15.4% of total cases. Infiltrations of CD4+, CD8+, and FOXP3+ TILs and PD-L1+ immune cells moderately correlated with one another (rho = 0.310~0.566; *p* < 0.001; Additional file 1: Table S1).

First, we evaluated the relationship between immune cell subset infiltration and clinicopathologic features of pure DCIS (Table 2). The high infiltration of CD4+, CD8+, and FOXP3+ TILs and the presence of PD-L1+ immune cells were commonly associated with high nuclear grade, comedo-type necrosis, ER negativity, PR negativity, and high Ki-67 index (all *p <* 0.05). In addition, the high infiltration of CD8+ and FOXP3+ TILs was associated with HER2 positivity (*p* = 0.010 and *p* = 0.004, respectively), and the infiltration of CD4+, CD8+, and FOXP3+ TILs was higher in tumors with p53 overexpression (*p* = 0.001, *p* = 0.041, and *p* = 0.016, respectively).

**Table 2:** Relationship between immune cell subset infiltration and clinicopathologic features of pure ductal carcinoma in situ

Clinicopathologic characteristics	CD4+ TIL		CD8+ TIL		FOXP3+ TIL		PD-L1+ IC	
	No. of TILs	***p*** value	No. of TILs	***p*** value	No. of TILs	***p*** value	Frequency (%)	***p*** value
Age (year)		0.577		0.218		0.815		0.792
< 50	19.7 (4.2–50.7)		12.7 (6.2–26.7)		0.0 (0.0–1.0)		21/132 (15.9)	
≥ 50	19.0 (3.8–44.5)		12.0 (4.2–22.2)		0.0 (0.0–1.0)		13/89 (14.6)	
DCIS extent (cm)		0.950		0.799		0.811		0.929
< 2.5	19.7 (5.0–47.5)		12.7 (5.3–24.2)		0.0 (0.0–1.0)		21/138 (15.2)	
≥ 2.5	17.3 (3.7–49.7)		11.3 (5.0–26.2)		0.0 (0.0–1.0)		13/83 (15.7)	
Nuclear grade		< 0.001		0.001		< 0.001		< 0.001
Low to intermediate	12.7 (2.0–37.5)		10.0 (4.7–18.2)		0.0 (0.0–0.0)		11/142 (7.7)	
High	25.3 (11.5–77.8)		15.7 (8.0–35.8)		0.0 (0.0–3.0)		23/79 (29.1)	
Comedo-type necrosis		0.004		0.002		0.008		< 0.001
Absent	16.0 (3.4–42.0)		10.7 (5.3–20.1)		0.0 (0.0–0.0)		17/174 (9.8)	
Present	31.5 (13.6–86.1)		22.5 (8.5–41.5)		0.0 (0.0–5.0)		17/47 (36.2)	
ER		0.026		0.025		0.004		0.043
Negative	34.0 (13.6–82.4)		22.5 (7.6–48.8)		0.5 (0.0–5.0)		8/27 (29.6)	
Positive	17.0 (3.7–43.5)		11.8 (5.3–22.0)		0.0 (0.0–0.0)		26/194 (13.4)	
PR		0.027		< 0.001		0.002		0.019
Negative	34.0 (12.1–76.3)		24.2 (10.2–48.9)		0.0 (0.0–4.9)		11/40 (27.5)	
Positive	16.0 (3.7–42.0)		10.7 (4.7–19.5)		0.0 (0.0–0.0)		23/181 (12.7)	
HER2		0.354		0.010		0.004		0.059
Negative	17.3 (3.8–44.5)		12.0 (5.2–22.0)		0.0 (0.0–0.0)		25/189 (13.2)	
Positive	28.7 (6.8–73.2)		22.0 (7.3–48.5)		0.0 (0.0–5.2)		9/32 (28.1)	
Ki-67 index		0.001		0.009		< 0.001		< 0.001
< 10%	15.8 (3.4–40.1)		11.0 (4.9–20.2)		0.0 (0.0–0.0)		17/164 (10.4)	
≥ 10%	34.8 (9.3–96.2)		16.2 (8.3–36.3)		0.0 (0.0–5.0)		17/57 (29.8)	
P53		0.001		0.041		0.016		0.092
Negative	16.3 (3.7–40.4)		11.5 (4.9–22.4)		0.0 (0.0–0.1)		26/192 (13.5)	
Positive	49.0 (18.8–80.8)		17.0 (9.4–31.2)		0.0 (0.0–3.0)		8/29 (27.6)	

For CD4+, CD8+, and FOXP3+ TILs, *p* values were calculated by the Mann-Whitney *U* test, and data are presented as median (interquartile range)

For PD-L1+ IC, *p* values were calculated by chi-square or Fisher’s exact test, and data are presented as frequency (%)

*TIL* tumor-infiltrating lymphocyte, *IC* immune cell, *DCIS* ductal carcinoma in situ, *ER* estrogen receptor, *PR* progesterone receptor, *HER2* human epidermal growth factor receptor 2

### Comparison of immune cell subsets between pure DCIS and invasive carcinoma

Next, we examined the difference in immune cell subset infiltration between pure DCIS and invasive carcinoma (Tables 3 and 4). When comparing pure DCIS and invasive carcinoma in the whole group, the infiltration of CD4+, CD8+, and FOXP3+ TILs and the presence of PD-L1+ immune cells were significantly higher in invasive carcinoma compared to pure DCIS (all *p* < 0.001; Table 3). In invasive breast cancer, TIL infiltration has been reported to be predominant in hormone receptor (HR)-negative tumors including HER2+ and triple-negative subtypes as opposed to HR-positive tumors [14, 25]. In this study, we found that the infiltration of immune cells was higher in HR-negative DCIS compared to HR-positive DCIS; thus, we performed subgroup analyses by HR status. In both HR-positive and HR-negative groups, all TIL subset infiltration and the presence of PD-L1+ immune cells were significantly higher in invasive carcinoma than in pure DCIS (all *p* < 0.001) (Table 3).

**Table 3:** Comparison of immune cell subset infiltration in pure ductal carcinoma in situ and invasive carcinoma

Hormone receptor status	Immune cell subset	Pure DCIS (***n*** = 231)	Invasive carcinoma (***n*** = 359)	***p*** value
Total	CD4+ TIL	19.3 (4.0–47.8)	85.5 (41.0–176.5)	< 0.001
	CD8+ TIL	12.3 (5.3–25.0)	91.5 (42.0–199.3)	< 0.001
	FOXP3+ TIL	0 (0–1.0)	9.0 (4.0–19.0)	< 0.001
	PD-L1+ IC	34/221 (15.4)	153/350 (43.7)	< 0.001
Positive	CD4+ TIL	17.0 (3.7–43.3)	79.0 (38.3–152.0)	< 0.001
	CD8+ TIL	11.7 (5.3–22.0)	68.5 (34.0–139.3)	< 0.001
	FOXP3+ TIL	0.0 (0.0–0.0)	6.5 (3.0–16.8)	< 0.001
	PD-L1+ IC	26/196 (13.3)	72/242 (29.8)	< 0.001
Negative	CD4+ TIL	35.0 (18.0–86.5)	125.0 (48.8–209.3)	< 0.001
	CD8+ TIL	22.7 (9.2–48.8)	160.0 (81.8–278.3)	< 0.001
	FOXP3+ TIL	1.0 (0.0–5.0)	15.0 (7.0–28.0)	< 0.001
	PD-L1+ IC	8/25 (32.0)	81/108 (75.0)	< 0.001

For CD4+, CD8+ and FOXP3+ TILs, *p* values were calculated by the Mann-Whitney *U* test, and data are presented as median (interquartile range)

For PD-L1+ IC*, p* values were calculated by chi-square or Fisher’s exact test, and data are presented as frequency (%)

*TIL* tumor-infiltrating lymphocyte, *IC* immune cell, *DCIS* ductal carcinoma in situ

**Table 4:** Comparison of CD4+ and CD8+ tumor-infiltrating lymphocyte infiltration in individual tumors

Disease group	Hormone receptor status	CD4+ TIL > CD8+ TIL	CD4+ TIL < CD8+ TIL	CD4+ TIL = CD8+ TIL	***p*** value
Pure DCIS	Total (*n* = 224)	135 (60.3)	88 (39.3)	1 (0.4)	< 0.001
	HR positive (*n* = 196)	116 (59.2)	79 (40.3)	1 (0.5)	< 0.001
	HR negative (*n* = 28)	19 (67.9)	9 (32.1)	0 (0.0)	0.016
Invasive carcinoma	Total (*n* = 358)	150 (41.9)	208 (58.1)	0 (0.0)	0.006
	HR positive (*n* = 248)	115 (46.4)	133 (53.6)	0 (0.0)	0.580
	HR negative (*n* = 110)	35 (31.8)	75 (68.2)	0 (0.0)	< 0.001

*p* values were calculated by the Wilcoxon signed rank test. Data are presented as number of cases (%)

*TIL* tumor-infiltrating lymphocyte, *DCIS* ductal carcinoma in situ

We also compared the dominance of CD4+ versus CD8+ TILs in pure DCIS and invasive carcinoma (Table 4). As a whole, the infiltration of CD4+ TILs was higher than that of CD8+ TILs in pure DCIS (*p* < 0.001), whereas the reverse was true in invasive carcinoma with the CD8+ TILs being the dominant subset (*p* = 0.006). In HR-positive tumors, while there was a higher infiltration of CD4+ T cells compared to CD8+ T cells in pure DCIS (*p* < 0.001), there was no difference in the amount of infiltration between the two TIL subsets in invasive carcinoma (*p* = 0.580). HR-negative tumors revealed the same pattern of TIL subset dominance as in the whole group.

### Comparison of immune cell subset infiltration in DCIS and invasive components in a tumor

**Fig. 2:**
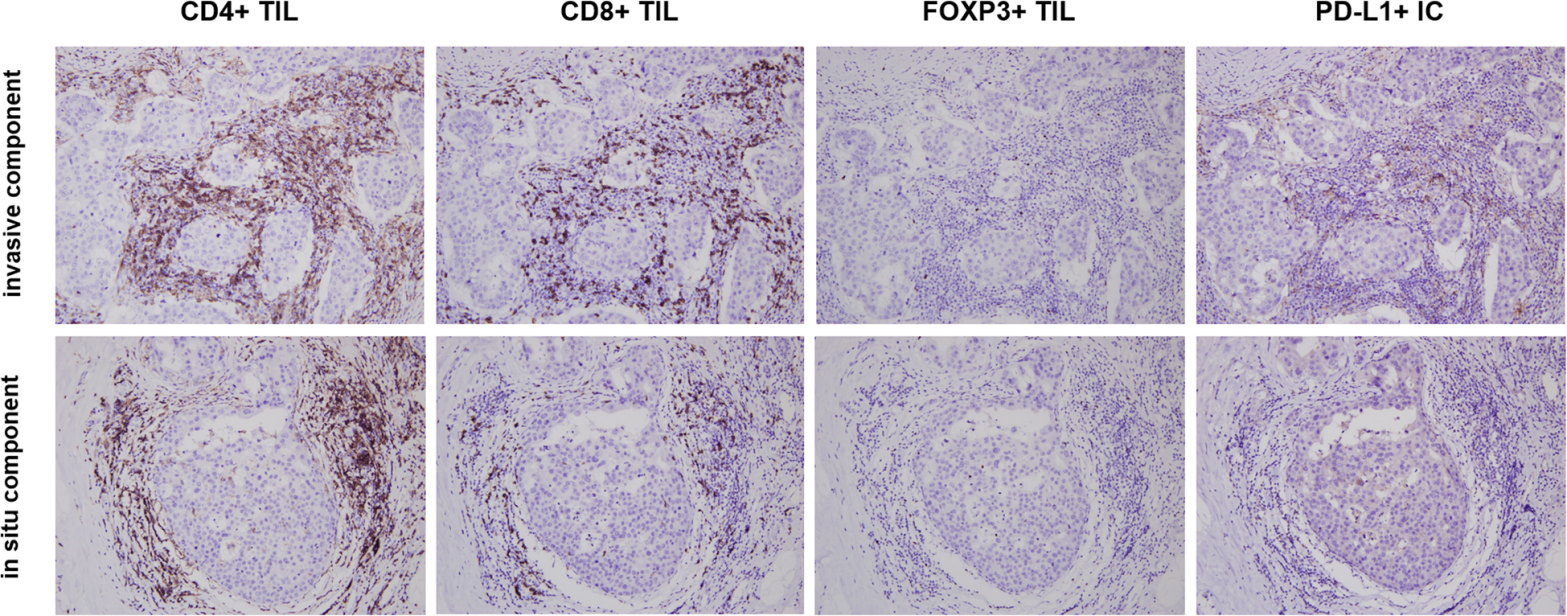
Representative example of CD4+, CD8+, FOXP3+ tumor-infiltrating lymphocytes and PD-L1+ immune cell infiltration in in situ and invasive components in a hormone receptor-negative tumor. CD8+ tumor-infiltrating lymphocyte (TIL) and PD-L1+ immune cell (IC) infiltration is significantly higher in the invasive component compared to the in situ component while CD4+ TIL infiltration is high in both components with no significant difference. FOXP3+ TILs are rarely found in both invasive and in situ components in this case

In order to evaluate the difference in TIL subset infiltration and presence of PD-L1+ immune cells between in situ and invasive components within individual tumors, we compared their infiltration in matched in situ and invasive components using 90 cases of invasive carcinoma with a DCIS component (Table 5; Fig. 2). All TIL subset infiltration and the presence of PD-L1+ immune cells were significantly higher in the invasive component compared to the in situ component (all *p <* 0.001). The HR-positive group revealed a similar pattern as the whole group with significant differences in the number of CD4+, CD8+, and FOXP3+ TILs, and presence of PD-L1+ immune cells between invasive and in situ components (*p <* 0.001, *p <* 0.001, *p <* 0.001, and *p* = 0.002, respectively). In the HR-negative group, CD8+ and FOXP3+ TILs and PD-L1+ immune cells were higher in the invasive component compared to the in situ component (*p =* 0.004, *p* = 0.022, and *p =* 0.031, respectively). However, there was no difference in CD4+ TIL infiltration between the two components (*p* = 0.584).

**Table 5:** Comparison of immune cell infiltration in the DCIS and invasive components of individual tumors

Hormone receptor status	Immune cell subset	Invasive component	DCIS component	Invasive > DCIS No. of cases	Invasive < DCIS No. of cases	Invasive = DCIS No. of cases	***p*** value
All (*n* = 90)	CD4+ TIL	79.5 (42.0–198.5)	38.5 (12.0–101.8)	69	26	0	< 0.001
	CD8+ TIL	58.5 (26.8–144.3)	18.0 (5.5–44.3)	71	18	1	< 0.001
	FOXP3+ TIL	4.0 (1.0–12.3)	1.0 (0.0–3.3)	56	16	18	< 0.001
	PD-L1+ IC	35 (38.9)	19 (21.1)	16^a^	0^b^	19^c^/55^d^	< 0.001
Positive (*n* = 67)	CD4+ TIL	73.0 (26.0–195.0)	18.0 (10.0–65.0)	49	18	0	< 0.001
	CD8+ TIL	45.0 (20.0–100.0)	11.0 (3.0–33.0)	53	13	1	< 0.001
	FOXP3+ TIL	2.0 (0.0–8.0)	1.0 (0.1–2.0)	41	8	18	< 0.001
	PD-L1+ IC	19 (28.4)	9 (13.4)	10^a^	0^b^	9^c^/48^d^	0.002
Negative (*n* = 23)	CD4+ TIL	124.0 (70.0–220.0)	101.0 (53.0–185.0)	15	8	0	0.584
	CD8+ TIL	143.0 (84.0–274.0)	45.0 (23.0–81.0)	18	5	0	0.004
	FOXP3+ TIL	10.0 (2.0–13.0)	4.0 (1.0–6.0)	15	8	0	0.022
	PD-L1+ IC	16 (69.6)	10 (43.5)	6^a^	0^b^	10^c^/7^d^	0.031

*p* values were calculated by the Wilcoxon signed rank test or McNemar test

Data are presented as median (interquartile range) for CD4+, CD8+, and FOXP3+ TILs and as number of positive cases (%) for PD-L1 IC

^a^Invasive (+)/DCIS (−); ^b^invasive (−)/DCIS (+); ^c^invasive (+)/DCIS (+); ^d^invasive (−)/DCIS (−)

*TIL* tumor-infiltrating lymphocyte, *IC* immune cell, *DCIS* ductal carcinoma in situ

### Comparison of immune cell subset infiltration in pure DCIS and DCIS-INV

We also determined the difference in infiltration of TIL subsets and PD-L1+ immune cells between pure DCIS, DCIS-M, and DCIS-INV (Table 6). When comparing pure DCIS and DCIS-M in the whole group, the infiltration of CD4+ and FOXP3+ TIL was significantly higher in DCIS-M than in pure DCIS (all *p <* 0.001). The comparison of pure DCIS and DCIS-INV revealed higher CD4+ and FOXP3+ TIL infiltrations in DCIS-INV than in pure DCIS (*p* = 0.004 and *p* = 0.005, respectively). As a whole, DCIS-M and DCIS-INV revealed no difference in immune cell infiltration.

**Table 6:** Comparison of the infiltration of immune cell subsets in pure DCIS, DCIS with microinvasion, and DCIS associated with invasive carcinoma

Hormone receptor status	Subset of immune cells	Pure DCIS (***n*** = 231)	DCIS-M (***n*** = 81)	DCIS-INV (***n*** = 90)	***p*** value			
					Three groups*	Pure DCIS vs. DCIS-M^**#**^	Pure DCIS vs. DCIS-INV^**#**^	DCIS-M vs. DCIS-INV^**#**^
All (*n* = 402)	CD4+ TIL	19.3 (4.0–47.8)	46.3 (16.7–112.1)	38.5 (12.0–101.8)	< 0.001	< 0.001	< 0.001	1.000
	CD8+ TIL	12.3 (5.3–25.0)	18.7 (6.3–47.8)	18.0 (5.5–44.3)	0.027	0.057	0.168	1.000
	FOXP3+ TIL	0 (0–1.0)	1.5 (0.0–10.0)	1.0 (0.0–3.3)	< 0.001	< 0.001	< 0.001	1.000
	PD-L1+ IC	34/221 (15.4)	20/78 (25.6)	19/90 (21.1)	0.111	0.129	0.669	1.000
Positive (*n* = 311)	CD4+ TIL	17.0 (3.7–43.3)	18.3 (6.7–85.7)	18.0 (10.0–65.0)	0.040	0.606	0.051	1.000
	CD8+ TIL	11.7 (5.3–22.0)	15.3 (5.2–34.7)	11.0 (3.0–33.0)	0.684	1.000	1.000	1.000
	FOXP3+ TIL	0.0 (0.0–0.0)	0.0 (0.0–2.0)	1.0 (0.1–2.0)	< 0.001	0.225	< 0.001	0.573
	PD-L1+ IC	26/196 (13.3)	6/38 (15.8)	9/67 (13.4)	0.916	1.000	1.000	1.000
Negative (*n* = 91)	CD4+ TIL	35.0 (18.0–86.5)	63.3 (31.7–115.0)	101.0 (53.0–185.0)	0.001	0.036	0.003	0.063
	CD8+ TIL	22.7 (9.2–48.8)	25.7 (9.0–64.0)	45.0 (23.0–81.0)	0.007	1.000	0.009	0.027
	FOXP3+ TIL	1.0 (0.0–5.0)	4.0 (1.0–12.0)	4.0 (1.0–6.0)	0.076	0.114	0.159	1.000
	PD-L1+ IC	8/25 (32.0)	14/40 (35.0)	10/23 (43.5)	0.690	1.000	1.000	1.000

*For the comparison of three groups, the Kruskal-Wallis test or chi-square test was used

^#^For the comparison of two groups, the Mann-Whitney *U* test or chi-square test was used. Corrections for multiple testing are performed with the Bonferroni method, and adjusted (adj.) *p* values are presented

Data are presented as median (interquartile range) for CD4+, CD8+, and FOXP3+ TILs and as frequency (%) for PD-L1+ IC

*TIL* tumor-infiltrating lymphocyte, *IC* immune cell, *DCIS* ductal carcinoma in situ, *DCIS-M* DCIS with microinvasion, *DCIS-INV* DCIS associated with invasive carcinoma

In HR-positive tumors, FOXP3+ TIL infiltration was significantly higher in DCIS-INV than in pure DCIS (*p* < 0.001) and CD4+ TIL infiltration tended to be higher in DCIS-INV than in pure DCIS (*p* = 0.051). In HR-negative tumors, CD4+ TIL showed a gradual increase from pure DCIS to DCIS-M and DCIS-INV (*p* = 0.036, pure DCIS vs. DCIS-M; *p* = 0.063, DCIS-M vs. DCIS-INV; *p* = 0.003, pure DCIS vs. DCIS-INV). CD8+ TIL infiltration was significantly higher in DCIS-INV compared to pure DCIS and DCIS-M (*p* = 0.009 and *p* = 0.027, respectively).

PD-L1+ immune cell infiltration revealed no difference between pure DCIS, DCIS-M, and DCIS-INV regardless of the HR status.

### Association of immune cell subset infiltration and patient outcome in pure DCIS

**Fig. 3:**
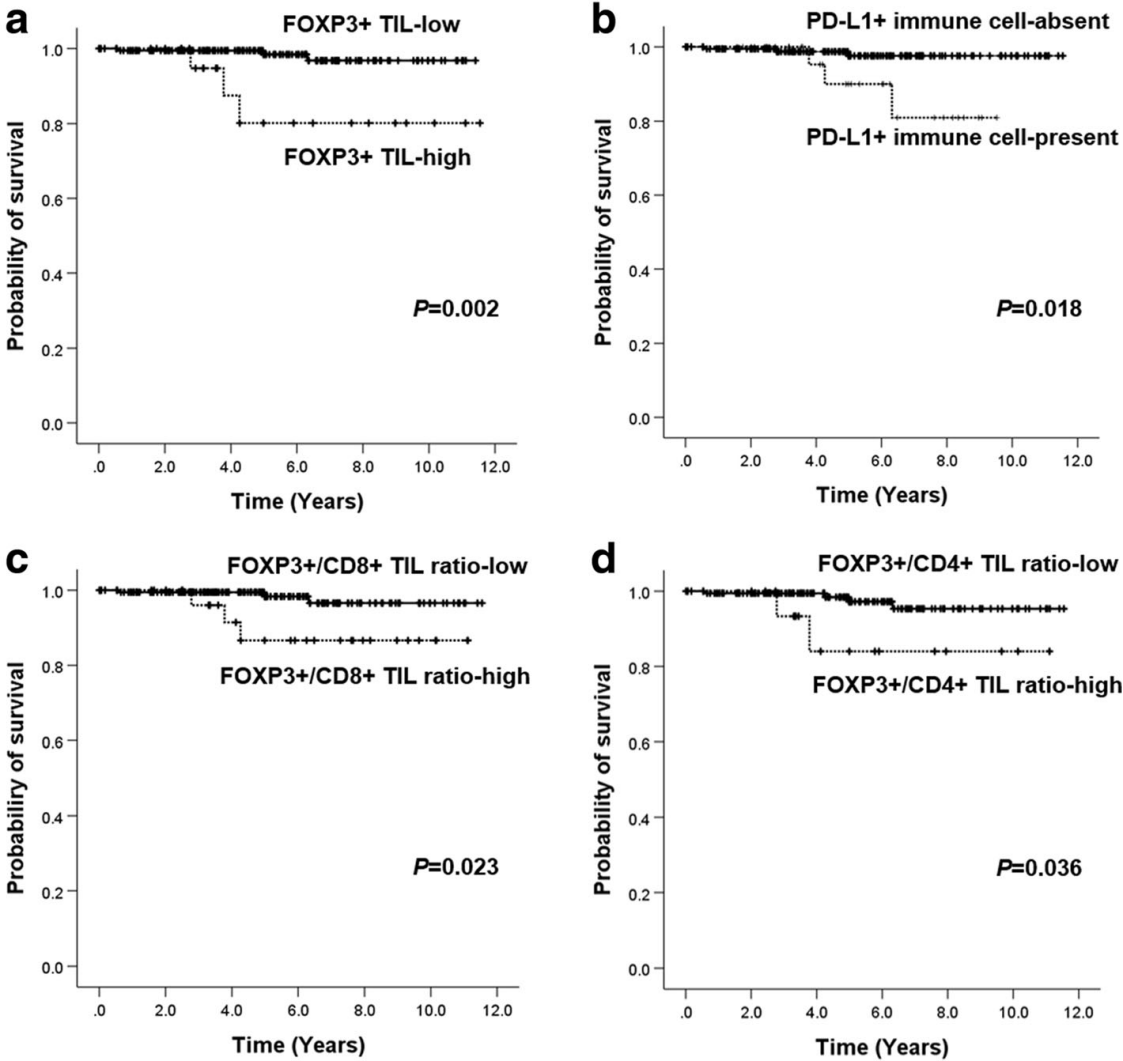
Kaplan-Meier survival curves stratified by immune cell subset infiltration and their ratio in pure DCIS. High level of FOXP3+ tumor-infiltrating lymphocyte (TIL) infiltration (**a**), the presence of PD-L1+ immune cells (**b**), high FOXP3+/CD8+ TIL ratio (**c**), and high FOXP3+/CD4+ TIL ratio (**d**) are associated with decreased recurrence-free survival

Finally, we evaluated the clinical outcome of the patients with pure DCIS in relation to immune cell subset infiltration. Most patients were treated according to standard guidelines and had been followed regularly after surgery. The median follow-up period was 4.7 years (range 0.1–11.5 years) during which 6 patients developed ipsilateral breast recurrence. Recurred tumors were pure DCIS in four patients and invasive ductal carcinoma in the remaining two patients. None of them had metastasis or cancer-related death thereafter. Other clinicopathologic characteristics of these 6 cases are summarized in Additional file 2: Table S2. In survival analyses, none of the clinicopathologic features (extent of DCIS, nuclear grade, comedo-type necrosis, HR status, HER2 status, Ki-67 index, p53 overexpression, and margin status) and therapies (type of surgery, adjuvant radiation therapy, and adjuvant endocrine therapy) was associated with ipsilateral breast recurrence. As for immune cell subset infiltration, the high infiltration of FOXP+ TILs and presence of PD-L1+ immune cells were found to be associated with decreased recurrence-free survival (*p* = 0.002 and *p* = 0.018, respectively; Fig. 3). However, CD4+ and CD8+ TIL infiltration did not show prognostic significance (*p* = 0.287 and *p* = 0.445, respectively, log-rank test). As the infiltration of TIL subsets was correlated with one another, we also analyzed the relationship between the ratios of TIL (FOXP3+/CD8+ TIL, FOXP3+/CD4+ TIL, and CD4+/CD8+ TIL) and tumor recurrence. High FOXP3+/CD8+ TIL ratio and high FOXP3+/CD4+ TIL ratio were associated with decreased recurrence-free survival (*p* = 0.023 and *p* = 0.036, respectively; Fig. 3).

Of the 6 cases with ipsilateral breast recurrence, 5 cases were HR positive. In subgroup analyses of HR-positive pure DCIS, the high infiltration of FOXP+ TILs and presence of PD-L1+ immune cells also revealed association with decreased recurrence-free survival (*p* = 0.019 and *p* = 0.002, respectively).

## Discussion

In this study, we have shown that the high infiltration of CD4+, CD8+, and FOXP3+ T cells and the presence of PD-L1+ immune cells were generally associated with aggressive features of DCIS including high nuclear grade, comedo-type necrosis, HR negativity, and high Ki-67 proliferation index. Increased TIL density in DCIS has been associated with high-risk features including large tumor size, high nuclear grade, comedo-type necrosis, ER negativity, and HER2 positivity in previous studies [15, 16, 26]. As TIL subset infiltration is higher in TIL-rich DCIS, it is reasonable to assume that high TIL subset infiltration is associated with aggressive features of DCIS. As for the relationship between TIL subset infiltration and characteristics of DCIS, a few studies have reported a positive correlation between TIL subset infiltration such as CD4+, CD8+, FOXP3+, and CD20+ cells and nuclear grade of DCIS [17, 27]. Similarly, the presence of PD-L1+ immune cells in DCIS has been reported to be associated with high TIL infiltration, younger patient age, ER negativity, and HER2 positivity in previous studies [16, 20–22].

Dense TIL infiltration in DCIS has been linked to a high risk of progression [15, 16]. As for the composition of TILs, low CD8+ T cells, low CD8+/FOX3+ T cell ratio, and low CD8+HLA-DR+ cells have been reported to be associated with ipsilateral recurrence [17, 19]; high numbers of B cells were associated with shorter recurrence-free interval in DCIS [18]. In this study, we showed that high infiltration of FOXP3+ TIL, high FOXP3+/CD8+ TIL ratio, and high FOXP3+/CD4+ TIL ratio were associated with decreased recurrence-free survival. Moreover, we also demonstrated that the presence of PD-L1+ immune cell was associated with poor recurrence-free survival. These results suggest that suppression of anti-tumor immunity by FOXP3+ TILs and PD-L1+ immune cells plays an important role during progression of DCIS. Thus, pure DCIS with high FOXP3+ TIL infiltration or PD-L1+ immune cells could be a target for active surveillance or aggressive treatment. However, the analyses about tumor recurrence have a limitation in that only a small number (*n* = 6) of cases revealed ipsilateral breast recurrence. Moreover, clinicopathologic variables which were known to be associated with recurrence in pure DCIS were not associated with tumor recurrence in this study. Thus, the results should be interpreted cautiously and further large studies are warranted to confirm these findings.

The evaluation of difference in immune cell infiltration between DCIS and invasive carcinoma and also among pure DCIS, DCIS-M, and DCIS-INV may provide some insight into its role during DCIS progression. We showed that all TIL subset infiltration and the presence of PD-L1+ immune cells were higher in invasive carcinoma than in pure DCIS irrespective of the HR status as in previous studies which reported a gradual increase in the number of immune cells during progression of breast cancer [28, 29]. Interestingly, in this study, HR-negative breast cancers revealed high CD4+ TIL infiltration in both in situ and invasive components of the same tumors with no statistical difference. Moreover, in HR-negative tumors, CD4+ TIL showed a gradual increase from pure DCIS to DCIS-M and DCIS-INV. These findings suggest that CD4+ T cells increase at an early stage of DCIS progression in HR-negative tumors and may play a crucial role during in situ to invasive transition in HR-negative tumors. However, we did not evaluate the CD4+ TILs by subset except for FOXP3+ regulatory T cells. The infiltration of FOXP3+ TILs did not differ between pure DCIS, DCIS-M, and DCIS-INV in the HR-negative group. It is well known that CD4+ TILs display a large degree of plasticity and the ability to differentiate into multiple sublineages in response to environmental cues [30]. CD4+ Th1 cells, CD4+ CTLs, and follicular helper T cells exert potent anti-tumor activity, whereas regulatory T cells or, under certain circumstances, CD4+ Th2 cells and CD4+ Th17 cells show tumor-promoting activity [30, 31]. Thus, in further studies, analyses of CD4+ TIL subsets would be crucial to find the overall effect of heavy CD4+ TIL infiltration around HR-negative DCIS.

In HR-positive breast cancers, despite the fact that FOXP3+ TIL was significantly higher in DCIS-INV than in pure DCIS, the other TIL subsets seemed to increase in number at a late stage of DCIS progression, or seemed unlikely to be associated with in situ to invasive transition. Growing evidence supports a role of host immune surveillance in influencing response to therapy and prognosis in HER2+ and triple-negative breast cancer, but not in HR-positive breast cancer which appears to be less immunogenic than HER2+ and triple-negative breast cancer [7]. However, it has been reported that FOXP3+ TIL infiltration is strongly associated with adverse clinical outcome in HR-positive breast cancer [14, 32, 33]. Furthermore, in the present study, we observed that infiltration of FOX3+ TIL was associated with tumor recurrence in HR-positive pure DCIS. Thus, treatment or prevention of tumor progression in HR-positive breast cancer would have to be focused on FOXP3+ regulatory T cells.

The mechanism by which immune cells influence tumor cell invasion in DCIS is unclear. Degradation of the basement membrane, a prerequisite for tumor invasion, has been attributed primarily to overproduction of proteolytic enzymes by the tumor or the surrounding stromal cells [34]. However, cumulating data support a hypothesis that myoepithelial cells act as “natural tumor suppressors” and lose such property during tumor progression [35]. Man et al. reported that leukocyte infiltration was increased at focal myoepithelial cell disruption sites in DCIS, suggesting that a localized death of myoepithelial cells and subsequent immune reactions are a trigger for myoepithelial cell layer disruptions, basement membrane degradation, and tumor invasion [36]. Conversely, it can be postulated that when tumor cells with an increased invasive property invade the stroma, it activates the immune system leading to more immune cell infiltration around DCIS with invasion.

We showed that CD4+ and FOXP3+ TIL infiltration was significantly higher in DCIS-M and DCIS-INV compared to pure DCIS in the whole group. CD4+ and CD8+ TIL infiltration was significantly higher in DCIS-INV than in pure DCIS in the HR-negative group, and FOXP3+ TIL infiltration was significantly higher in DCIS-INV than in pure DCIS in the HR-positive group. Beguinot et al. reported that microinvasive carcinoma showed a significantly higher TIL density with more CD8+, CD4+, and CD38+ cell infiltration than pure DCIS. Toss et al. also showed that DCIS-INV showed denser TILs as opposed to pure DCIS [15]. Although these authors did not show the difference in TIL infiltration according to HR status, their findings are consistent with the results of our study. It is known that in situ and invasive components of the same tumor exhibit similar patterns of genetic alterations [37]. Thus, tumor cells in DCIS-INV may show higher immunogenicity than tumor cells in pure DCIS eliciting stronger immune responses compared to pure DCIS.

In our study, there were more CD4+ T cells than CD8+ T cells infiltrating pure DCIS regardless of the HR status. In invasive carcinoma, CD4+ and CD8+ T cell infiltration showed no difference in HR-positive tumors, whereas CD8+ T cells were predominant in HR-negative tumors. In line with our study, Thompson et al. found slightly more CD4+ T cells than CD8+ T cells in DCIS [20], and Sheu et al. showed that CD8+ T cells significantly increased with stage progression of invasive breast cancer [38]. On the contrary, Gil Del Alcazar et al. [39] reported a decrease in CD8+ signatures in invasive breast cancer and fewer activated GZMB+CD8+ T cells in invasive breast cancer compared to DCIS. However, these studies were conducted using a small series, and we observed a greater infiltration of CD8+ T cells than CD4+ T cells in a minority of DCIS, and vice versa in some cases of invasive carcinoma. Thus, further large-series studies investigating the composition of TIL subsets in DCIS and their change during tumor progression are required to understand the role of individual TIL subsets during tumor progression.

The current study includes a relatively large number of cases that can provide a general idea on changes in immune cell infiltration during in situ to invasive transition. However, this study has some limitations. First, even though the International Immuno-Oncology Biomarker Working Group recently published a proposal for evaluation of TILs on hematoxylin- and eosin-stained sections in DCIS [23, 24], the scoring system for TIL subsets by immunohistochemistry in DCIS has not been optimized yet. It is recommended that only stromal TILs be evaluated as mean values in DCIS [23, 24]. However, we evaluated both intra-tumoral and stromal TIL subsets in hot spots in DCIS to compare with our previous study data of TIL subset infiltration in invasive carcinoma even though most of the TIL subsets were found in the stromal compartment. Second, the evaluation of immune cell subsets was confined to CD4+, CD8+, and FOXP3+ TILs and PD-L1+ immune cells, and their subtypes or activation status was not assessed. In addition, although TIL subset and PD-L1+ immune cell infiltration in DCIS may be heterogeneous from one area to another, the cells were counted using the TMA platform in order to evaluate a large number of samples which may have resulted in selection bias.

## Conclusion

In summary, we showed that the immune microenvironment of DCIS is different from that of invasive carcinoma. CD4+, CD8+, and FOXP3+ TIL and PD-L1+ immune cell infiltration was significantly higher in invasive carcinoma compared to pure DCIS regardless of the HR status. In HR-negative tumors, there was no difference in CD4+ TIL infiltration between in situ and invasive components within the same tumors, and it increased in a stepwise fashion from pure DCIS to DCIS-M and DCIS-INV, indicating its possible role during early stage of HR-negative DCIS progression. In HR-positive tumors, all immune cell infiltrations were higher in the invasive component than in the DCIS component, and FOXP3+ TIL was significantly higher in DCIS-INV than in pure DCIS. Finally, the high infiltration of FOXP3+ TIL and the presence of PD-L1+ immune cells were associated with tumor recurrence in patients with pure DCIS, suggesting a potential benefit in active surveillance or aggressive treatment in such patient groups.

## Supplementary information

**Additional file 1: Table S1.** Correlations in infiltration of CD4+, CD8+, and FOXP3+ tumor infiltrating lymphocytes and PD-L1+ immune cells in pure ductal carcinoma in situ.

**Additional file 2: Table S2.** Summary of 6 cases with ipsilateral breast recurrence.

## Data Availability

The datasets used and/or analyzed during the current study are available from the corresponding author on reasonable request.

## Abbreviations

DCIS: Ductal carcinoma in situ
DCIS-M: DCIS with microinvasion
DCIS-INV: DCIS associated with invasive carcinoma
ER: Estrogen receptor
HER2: Human epidermal growth factor receptor 2
HR: Hormone receptor
TIL: Tumor-infiltrating lymphocyte
PR: Progesterone receptor
