# Supplementary material for: Immune microenvironment in ductal carcinoma in situ: a comparison with invasive carcinoma of the breast
Source: Breast Cancer Res. 2020 Mar 26;22:32. doi: 10.1186/s13058-020-01267-w (PMC7098119; doi:10.1186/s13058-020-01267-w)
Supplement: Supplementary file 1 — Additional file 1: Table S1. Correlations in infiltration of CD4+, CD8+, and FOXP3+ tumor infiltrating lymphocytes and PD-L1+ immune cells in pure ductal carcinoma in situ. [file 13058_2020_1267_MOESM1_ESM.pdf]

Table S1. Correlations in infiltration of CD4+, CD8+, and FOXP3+ tumor infiltrating lymphocytes and PD-L1+ immune cells in pure ductal carcinoma in situ

Correlation between markers	CD4+ TIL	CD8+ TIL	FOXP3+ TIL	PD-L1+ IC
CD4+ TIL	-	0.566 (<0.001)	0.471 (<0.001)	0.404 (<0.001)
CD8+ TIL	0.566 (<0.001)	-	0.418 (<0.001)	0.310 (<0.001)
FOXP3+ TIL	0.471 (<0.001)	0.418 (<0.001)	-	0.417 (<0.001)
PD-L1+ IC	0.404 (<0.001)	0.310 (<0.001)	0.417 (<0.001)	-

Data are presented as rho correlation coefficients calculated by Spearman’s rank correlation test and p values in parentheses.

TIL, tumor-infiltrating lymphocyte; IC, immune cell
