# Supplementary material for: Immune microenvironment in ductal carcinoma in situ: a comparison with invasive carcinoma of the breast
Source: Breast Cancer Res. 2020 Mar 26;22:32. doi: 10.1186/s13058-020-01267-w (PMC7098119; doi:10.1186/s13058-020-01267-w)
Supplement: Supplementary file 2 — Additional file 2: Table S2. Summary of 6 cases with ipsilateral breast recurrence. [file 13058_2020_1267_MOESM2_ESM.pdf]

Table S2. Summary of 6 cases with ipsilateral breast recurrence

Serial No.	Extent of DCIS (cm)	Nuclear grade	HR	HER2	Node status	Surgery	Safety margin (cm)	Adjuvant radiation therapy	Adjuvant endocrine therapy	Time to recurrence (year)	Type of recurred tumor
10	1.5	2	+	-	Nx	BCS	0.5	+	-	4.96	DCIS
23	2.0	3	-	-	Nx	BCS	0.4	+	-	2.79	IDC
41	1.6	3	+	-	Nx	BCS	1.0	+	-	4.24	DCIS
45	4.0	2	+	-	Nx	BCS	<0.1	+	+	6.32	IDC
81	2.7	2	+	-	Nx	BCS	0.9	+	+	3.78	DCIS
166	0.8	2	+	-	Nx	BCS	2.0	+	+	0.63	DCIS

HR, hormone receptor; BCS, breast conserving surgery; DCIS, ductal carcinoma in situ; IDC, invasive ductal carcinoma
